# Reducing l‐lactate release from hippocampal astrocytes by intracellular oxidation increases novelty induced activity in mice

**DOI:** 10.1002/glia.23960

**Published:** 2021-01-05

**Authors:** Barbara Vaccari Cardoso, Alexey V. Shevelkin, Chantelle Terrillion, Olga Mychko, Valentina Mosienko, Sergey Kasparov, Mikhail V. Pletnikov, Anja G. Teschemacher

**Affiliations:** ^1^ School of Physiology Pharmacology and Neuroscience, University of Bristol Bristol UK; ^2^ Department of Psychiatry and Behavioral Sciences Johns Hopkins University School of Medicine Baltimore Maryland USA; ^3^ Institute of Biomedical and Clinical Sciences College of Medicine and Health, University of Exeter Exeter UK; ^4^ Solomon H. Snyder Department of Neuroscience Johns Hopkins University School of Medicine Baltimore Maryland USA; ^5^ Department of Physiology and Biophysics University at Buffalo New York New York USA

**Keywords:** astrocytes, behavior, hippocampus, lactate oxidase, lentiviral vector, l‐lactate, novelty

## Abstract

Astrocytes are in control of metabolic homeostasis in the brain and support and modulate neuronal function in various ways. Astrocyte‐derived l‐lactate (lactate) is thought to play a dual role as a metabolic and a signaling molecule in inter‐cellular communication. The biological significance of lactate release from astrocytes is poorly understood, largely because the tools to manipulate lactate levels in vivo are limited. We therefore developed new viral vectors for astrocyte‐specific expression of a mammalianized version of lactate oxidase (LOx) from *Aerococcus viridans*. LOx expression in astrocytes in vitro reduced their intracellular lactate levels as well as the release of lactate to the extracellular space. Selective expression of LOx in astrocytes of the dorsal hippocampus in mice resulted in increased locomotor activity in response to novel stimuli. Our findings suggest that a localized decreased intracellular lactate pool in hippocampal astrocytes could contribute to greater responsiveness to environmental novelty. We expect that use of this molecular tool to chronically limit astrocytic lactate release will significantly facilitate future studies into the roles and mechanisms of intercellular lactate communication in the brain.

## Main Points

- Lactate oxidase (LOx) expression limits the intra‐astrocytic pool of lactate and decreases its release.
- LOx in astrocytes of the dorsal mouse hippocampus causes increased activity in novel environment.

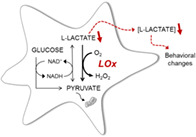

## 1 INTRODUCTION

Astrocytes are the major regulators of brain metabolic homeostasis as they are able to import from the periphery, metabolize, store and dynamically shuttle energy substrates throughout the astrocytic network and into the brain's extracellular space (Marina et al., 2020; Verkhratsky, Nedergaard, & Hertz, 2015; Weber & Barros, 2015). At the same time, astrocytes, by responding to local neuronal activity and the extracellular environment, actively participate in synaptic transmission and inter‐cellular communication (Gourine et al., 2010; Halassa, Fellin, & Haydon, 2007; Lalo et al., 2014; Mastitskaya et al., 2020). Astrocyte‐derived l‐lactate (lactate), in particular, has gained increasing attention due to its dual role as a metabolic and signaling molecule in the brain (Barros & Weber, 2018; Karagiannis et al., 2015; Magistretti & Allaman, 2018; Marina et al., 2018, 2020; Mosienko et al., 2018; Mosienko, Teschemacher, & Kasparov, 2015).

During periods of increased local brain activity, astrocytes elevate their release of lactate to support a wide range of brain area‐dependent functions. Examples include promotion of learning and memory in hippocampus and cortex (Alberini, Cruz, Descalzi, Bessières, & Gao, 2018; Newman, Korol, & Gold, 2011; Yang et al., 2014; Zuend et al., 2020), cerebral angiogenesis (Morland et al., 2017), regulation of sympatho‐excitation in the ventro‐lateral medulla (Marina et al., 2015), improvement of decision making behavior by synchronization of amygdala with anterior cingulate cortex (Wang et al., 2017), and amplification of the arousal response evoked by the *locus coeruleus* (Barros & Weber, 2018; Mosienko et al., 2018; Tang et al., 2014). Lactate is also known to modulate mood by as yet unknown mechanisms of action. Acute or chronic peripheral administration of lactate in animal models of depression resulted in an antidepressant‐like effect (Carrard et al., 2018; Jouroukhin et al., 2018; Karnib et al., 2019). This response was accompanied by increased levels of lactate in the hippocampus as well as changes in the expression of genes associated with serotonin receptor trafficking, the astrocytic calcium‐binding protein S100β, neurogenesis and cAMP signaling (Carrard et al., 2018).

However, the mechanisms whereby lactate influences metabolic and signaling pathways are poorly understood as the tools to manipulate lactate levels in vivo in a cell type‐specific manner are limited. With this in mind, we developed a novel viral vector system to express a mammalianized version of lactate oxidase (LOx) from *Aerococcus viridans* under control of an enhanced astrocyte‐specific promoter (Liu et al., 2008). Expression of LOx in primary astrocytes reduced their intracellular lactate pool and limited the release of lactate. In comparison to control mice, mice selectively expressing LOx in astrocytes of the hippocampus demonstrated increased responsiveness to novel environmental stimuli.

## 2 MATERIALS AND METHODS

### 2.1 Viral vector construction

The sequence of lactate oxidase (LOx; E.C. 1.1.3.15) from *Aerococcus viridans* (GenBank: D50611.1) was optimized for mammalian codon usage and synthesized by Invitrogen (Thermo Fisher Scientific UK).

For functional testing in HEK293 cells, we constructed pCMV‐LOx‐IRES‐EGFP, an expression vector with CMV promoter where the LOx open reading frame was inserted in 5′‐position of an internal ribosomal entry site (IRES), followed by green fluorescent protein (EGFP; Figure S1a). The equivalent cassette devoid of LOx served as control plasmid (pCMV‐IRES‐EGFP). Cells were transfected with TransIT‐293 (Mirus, MIR 2700) 2–3 days prior to experiments.

For in vitro tests in primary astrocytes, an adenoviral vector (AVV), AVV‐sGFAP‐LOx‐IRES‐tdTomato (Figure S1b), was constructed by standard homologous recombination (Bett, Haddara, Prevec, & Graham, 1994; Duale et al., 2005). In order to limit expression to astrocytes, we employed a transcriptionally enhanced shortened human GFAP promoter (sGFAP) (Liu et al., 2008). TdTomato was adopted as a reporter gene to avoid interference with the emission spectra of concomitantly used FRET sensor. Where indicated, AVV‐sGFAP‐EGFP was used as control.

For gene delivery into astrocytes in vivo, the expression cassette was transferred from AVV to a lentiviral vector (LVV), LVV‐sGFAP‐LOx‐IRES‐tdTomato (Figure S1c) (Coleman et al., 2003; Duale et al., 2005; Teschemacher, Paton, & Kasparov, 2005). LVV‐sGFAP‐IRES‐tdTomato was created as a control vector.

### 2.2 Primary cultures

Neonate Wistar rats were used in accordance with Schedule 1 of the UK Home Office (Scientific Procedures) Act (1986) and as approved by the University of Bristol ethics committees. Primary astrocytes were cultured following protocols adapted from (Marriott, Hirst, & Ljungberg, 1995). Brains were dissected from Wistar P2 pups, and the coronal section including midbrain and brainstem was dissociated by trituration following incubation in Hank's Balanced Salt Solution (HBSS) with DNase I (0.04 mg/ml), BSA (3 mg/ml), and bovine pancreas trypsin (0.25 mg/ml). Cells were incubated (37°C; 5% CO_2_) in culture media (Dulbecco's Modified Eagle Medium, 10% heat‐inactivated fetal bovine serum, 100 U/ml penicillin, 0.1 mg/ml streptomycin) for 7 days. Flasks were gently shaken overnight to eliminate microglia and oligodendrocytes and astrocytes were passaged. Following another 7 days in culture, AVVs were added to culture media 2–3 days prior to experiments to allow for optimal transgene expression.

Preparation of organotypic slice cultures followed the previously described protocol (Teschemacher et al., 2005). The brainstem was isolated from P7‐8 Wistar pups and transferred to sterile dissection medium containing HBSS, 20 mM glucose, 10 mM MgCl2, 1 mM HEPES, 1 mM kynurenic acid, 0.5% phenol red, 100 U/ml penicillin and 0.1 mg/ml streptomycin (4°C). A vibrating microtome (7,000 SMZ, Campden Instruments, Loughborough, UK) was used to cut 200‐μm‐thick coronal slices. Slices were deposited on culture membranes (Millicell‐CM, Millipore) in six‐well plates with media consisting of 49% Opti‐MEM with GlutaMAX (Life Technologies, 51985026), 25% HBSS, 25% fetal bovine serum, 1% penicillin (10,000 units)/streptavidin (10 ml/L), 25 mM glucose. Slices were transduced with ~10^9^ TU/ml of AVV‐sGFAP‐LOx‐IRES‐tdTomato for expression of LOx specifically in astrocytes and kept at 37°C and 5% CO2 for 8–12 days prior to experimentation.

### 2.3 Cell viability assays

Primary astrocytes were exposed to a range of AVV‐sGFAP‐LOx‐IRES‐tdTomato titers, in order to determine the optimal multiplicity of infection (MOI) and avoid non‐specific toxicity arising from overexpression in astrocytes. For the Trypan Blue exclusion assay, astrocytes were harvested by trypsinization and stained with trypan blue solution 0.4% (Sigma, T8154). The percentage of blue non‐viable cells was determined. The XTT (2,3‐bis‐(2‐methoxy‐4‐nitro‐5‐sulfophenyl)‐2H‐tetrazolium‐5‐carboxanilide) cell viability assay was performed according to the manufacturer's instructions (Cell Signaling Technology, 9095). The formazan absorbance after 2 hr, which is proportional to the number of metabolically viable cells, was read at 450 nm in a microplate reader (Tecan Infinite M200 PRO, Labtech). For estimation of reactive oxygen species (ROS), the H_2_DCFDA (2',7'‐dichlorodihydro‐fluorescein diacetate) assay was used. Astrocytes were washed twice with HBSS and the baseline fluorescence intensity was recorded with a fluorescence microscope (Zoe Cell Imager, Bio‐Rad; excitation: 480/17 nm, emission: 517/23 nm). Astrocytes were then incubated with H_2_DCFDA [5 μM] for 30 min at 37°C. The loading buffer was removed and the astrocytes were washed with HBSS, followed by immediate recording of the fluorescence intensity. For a positive control, astrocytes were exposed to H_2_O_2_ for 10 min. Fluorescence signal after dye exposure was normalized to baseline fluorescence.

### 2.4 Measurements of extracellular lactate in vitro

Constitutive release of lactate into culture media was determined with a fluorometric assay (EnzyFluo l‐lactate Assay Kit, BioAssay Systems, EFLLC‐100) following the manufacturer's instructions. Media were exchanged and subsequently sampled after 2 hr for HEK293 and 6 hr for astrocytes. Following the enzymatic reaction, fluorescence was determined in the microplate reader with 530 nm excitation at 585 nm emission. The lactate concentrations were determined according to the lactate standard curve.

Real‐time measurement of lactate release in organotypic slices was performed using amperometric lactate biosensors (Sarissa Biomedical, UK, SBS‐LAC‐05‐50, SBI‐NUL‐05‐50). Sensors were calibrated by applying defined concentrations of lactate to the chamber before and after each recording. Slices were transferred to the recording chamber and continuously superfused with HEPES‐buffered solution (HBS; in mM: NaCl 137, KCl 5.4 or 3, Na_2_HPO_4_ 0.34, KH_2_PO_4_ 0.44, CaCl_2_ 1.6, MgSO_4_ 0.8, NaHCO_3_ 4.2, HEPES 10, Glucose 5.5; pH 7.4) at 2.5 ml/min (32.5 ± 1°C). The electrode potential was controlled by a potentiostat (Duo‐Stat ME200+) and the signal was processed and analyzed using a 1,401 interface and Spike 2 software (Cambridge Electronic Design, UK). Electrodes were brought to contact with the slice surface and left to stabilize for at least 30 min. Sensors were then moved to a different area of the slide and allowed to plateau following the initial contact, before measurements of the constitutive release (lactate tone) were taken.

For forced lactate efflux from the cells, a trans‐acceleration protocol was employed by delivering 10 mM pyruvate to the recording chamber (Mächler et al., 2016; San Martín et al., 2013; Zuend et al., 2020). This approach relies on the inward gradient of the monocarboxylate transporter (MCT) substrate pyruvate to trigger MCT‐mediated lactate extrusion from the cells (Fishbein, Foellmer, Davis, Fishbein, & Armbrustmacher, 1988).

### 2.5 Assessing intracellular lactate in vitro

Primary cultures of astrocytes on collagenized glass coverslips were transduced with an AVV to express the lactate FRET sensor Laconic (San Martín et al., 2013) and co‐transduced with AVV‐sGFAP‐LOx‐IRES‐tdTomato. Coverslips were transferred to a recording chamber mounted on an upright Leica confocal microscope (SP1 or SP5) and continuously superfused with HBS (glucose 2 mM) at 2.5 mL/min (32.5 ± 1°C). Laconic was excited with 458 nm and emission bands for mTFP and Venus were sampled every 3 s for 465–500 nm and 515–595 nm bands, respectively. Regions of interest were analyzed for the mTFP/Venus FRET ratio over time and average changes after pyruvate application were normalized to the baseline.

### 2.6 In vivo experiments

#### 2.6.1 Animals and surgical procedures

C57Bl/6J mice (female, 8 weeks of age) were obtained from Jackson Lab and housed in groups of 3–5 animals in standard mouse cages on a 12‐hr light/dark cycle at a room temperature of 23°C with free access to food and water. The animal protocols were carried out in accordance with the Johns Hopkins University Animal Care and Use Committee. All experiments were performed during the light half of the cycle. Procedures were conducted using aseptic techniques. Mice were anesthetized with a 0.75–1% isoflurane‐oxygen mixture (2% for induction) and placed in a supine position on a heating pad connected to a rectal thermometer (∼37°C). For the dorsal hippocampus, 0.5 μl of LVV‐sGFAP‐IRES‐tdTomato (control; 5 × 10^9^ TU/ml) or LVV‐sGFAP‐LOx‐IRES‐tdTomato (7 × 10^9^ TU/ml) were injected bilaterally into the dorsal CA1 area of the hippocampus (AP, ML, DV: −1.9 mm; ±1.4 mm; −1.6 mm) at 1 nl/s using Nanoject III (Drummond Scientific Company, Broomall, PA), as previously described (Jouroukhin et al., 2019). After the injection, we waited 10 min before raising the glass micropipette. After surgery, mice were treated with buprenorphine (0.01 mg/kg s.c.) and Baytril (enrofloxacin, 2.5 mg/kg).

#### 2.6.2 Behavioral tests

Mice were tested 4 weeks following surgery in a series of behavioral tests to assess novelty‐induced activity in the open field, anxiety in the elevated plus maze (EPM), and exploratory activity in the hole board test. For the hole board test, the mice were placed in the center of a 40 cm × 40 cm hole board enclosure, with 16 three‐centimeter holes spaced evenly (Stoelting Co., IL). Mice were allowed to explore the hole board for 5 min. Distance traveled and head dips were recorded using any‐maze tracking software (Stoelting, Co.). These tests were followed by those for learning and memory: spontaneous alternation and spatial recognition in the Y maze, the novel object recognition test (NORT) and novel place recognition test (NPRT) and trace fear conditioning as previously described in (Abazyan et al., 2010, 2014; Jouroukhin et al., 2018; Pletnikov et al., 2008; Terrillion et al., 2017). All tests were conducted in the order that minimized potential carry‐over effects and stressful experience confounds, namely, with a one‐week interval between tests and from less to more stressful tests.

#### 2.6.3 Immunohistochemistry

Transgene expression in primary cultured astrocytes and organotypic brainstem slices was confirmed by immunoreactivity to red fluorescent protein (RFP). Cells were fixed with ice‐cold 4% paraformaldehyde (PFA) in 0.1 M phosphate buffer (PBS; pH 7.4), rinsed with PBS and incubated in a blocking and permeabilizing solution for 1 hr at room temperature (RT), followed by incubation with the anti‐RFP primary antibody (1:200, Table S1) overnight at 4°C. An Alexa Fluor 594‐labeled species‐specific secondary antibody was used. Images were obtained with an upright Leica SP5 confocal laser‐scanning microscope with ×25 or ×40 water immersion objective lenses.

Upon completion of behavioral tests, mice were deeply anesthetized with Forane (isoflurane USP, NDC 10019–360‐60, Baxter Healthcare Corporation, Deerfield, IL), followed by transcardial perfusion with ice‐cold 0.1 M PBS and 4% PFA in 0.1 M PBS. The brains were dissected out and post‐fixed in 4% PFA in 0.1 M PBS for 24 hr at 4°C. After cryoprotection in 30% sucrose in 0.1 M PBS for 48 hr, the brains were cut into 40‐μm thick coronal sections.

The transduced brain areas were identified by their expression of tdTomato. To evaluate the brain cell types that were transduced with LVV, brain sections were stained with chicken anti‐GFAP (1:1,000), rabbit anti‐S100 beta (1:1,000), rabbit anti‐Iba1 (1:1,000) or Guinea pig anti‐NeuN (1:1,000) antibodies as previously described (Hoffman, Murphy, & Sita, 2016; Jouroukhin et al., 2019). Briefly, after incubating brain sections in the blocking solution for 1 hr at RT, the sections were incubated for 48 hr at 4°C with the primary antibodies. Afterwards, the sections were incubated for 2 hr at RT with the corresponding Alexa 488‐, 568‐, 633‐labeled species‐specific secondary antibodies (1:1,000) followed by three 5‐min 3 x PBS washes and DAPI 10 min staining (1:10,000). Images were taken with a Zeiss 880 confocal laser‐scanning microscope with ×40/1.3 oil DIC or ×100/1.46 oil DIC objectives at the Johns Hopkins University Neuroscience Multiphoton/Electrophysiology Core Facility.

### 2.7 Statistical analysis

Data were subjected to a Shapiro–Wilk normality test. Groups were compared using paired or unpaired two‐tails *t*‐tests, ANOVA followed by Bonferroni's Multiple Comparison post hoc test, or non‐parametric Wilcoxon‐Mann–Whitney *U* test, as indicated. Box‐and‐whisker diagrams illustrate the upper and lower quartile values (boxes), median (central horizontal line), mean (central plus sign), and minimum and maximum data range (whiskers). Statistical tests were performed using GraphPad Prism. Statistical difference of *p* < 0.05 between the experimental groups was considered significant.

## 3 RESULTS

### 3.1 In vitro effects of LOx expression

**FIGURE 1:**
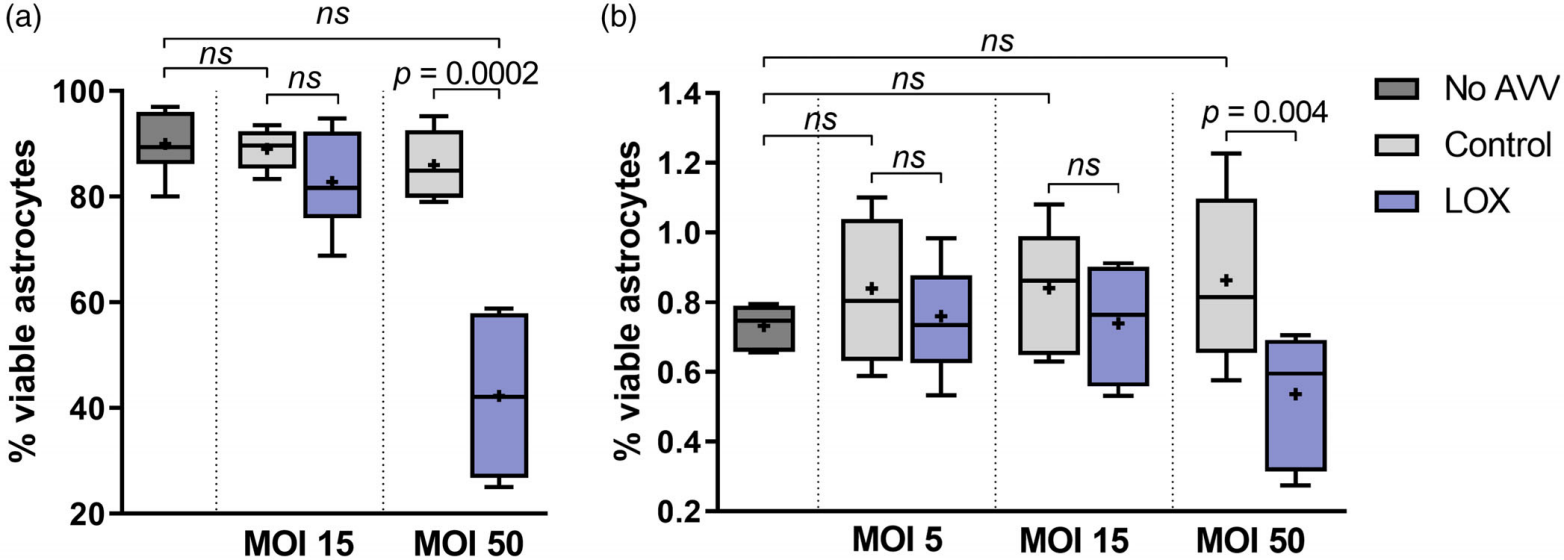
In vitro viability of adenoviral vector (AVV)‐transduced cultured astrocytes expressing lactate oxidase (LOx) or a control reporter. Trypan Blue exclusion assay (a) and XTT assay (b) consistently reported no effect of AVV‐sGFAP‐LOx‐IRES‐tdTomato on cell viability below an AVV MOI of 50; cell viability was unaffected by transduction with reporter (EGFP) expressing control AVV across the range of tested MOI ratios. (a) Sample sizes *n* = 8 wells in No AVV, *n* = 5 and *n* = 6 wells in Control MOI 15 and 50, respectively, and *n* = 6 and *n* = 5 wells in LOx MOI 15 and MOI 50, respectively. (b) Sample sizes *n* = 6 wells in no AVV, and *n* = 9 wells in Control and LOx at all MOIs tested. ANOVA (Bonferroni's Multiple Comparison Test) for comparison between control AVV transduction and un‐transduced astrocytes; unpaired two‐tailed *t*‐test for comparison of LOx to control reporter expressing astrocytes at each MOI tested [Color figure can be viewed at wileyonlinelibrary.com]

Astrocytes transduced with AVV‐sGFAP‐LOx‐IRES‐tdTomato at MOI 15 in dissociated primary cultures and with 10^9^ TU/ml in organotypic slices displayed typical in vitro morphology and their survival was not compromised compared to un‐transduced cultures (Figure S2b,c). A range of MOIs of AVV‐sGFAP‐LOx‐IRES‐tdTomato was used to transduce cultures, and their viability was assessed by Trypan Blue exclusion assay and XTT assay. In neither assay did control vector AVV‐sGFAP‐EGFP compromise the viability of astrocytes below an MOI of 50 (Figure 1). Therefore, all the functional validation experiments were carried out at MOI 15. LOx‐expressing astrocytes did not show increased concentration of intracellular ROS, suggesting that astrocytes accommodate the hydrogen peroxide produced by LOx activity (Figure S3).

### 3.2 LOx expression reduces constitutive lactate release in vitro

**FIGURE 2:**
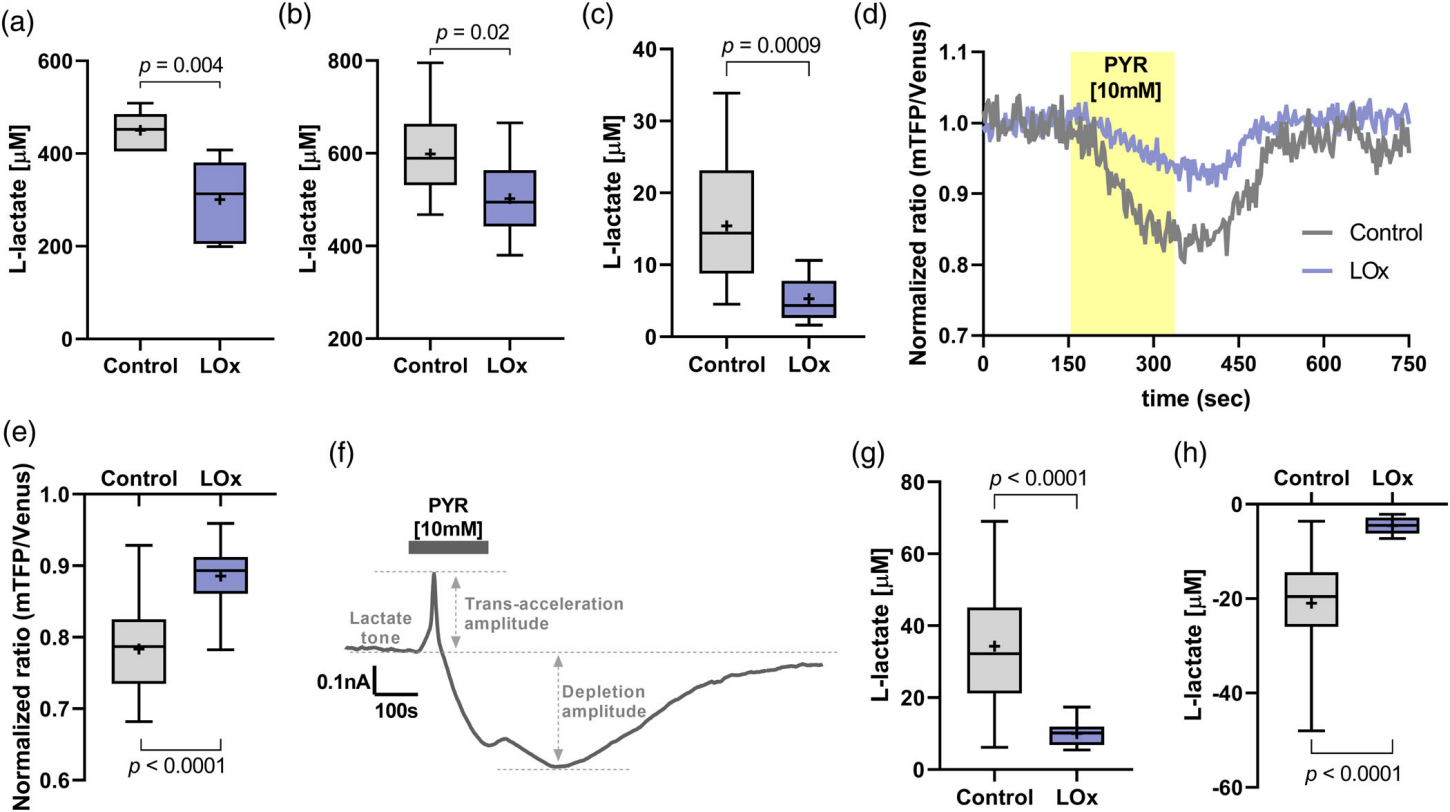
Lactate oxidase (LOx) expression reduces lactate release in vitro. (a) Media conditioned for 2 hr by HEK293 cells transfected with 1 μg/μl of either pCMV‐IRES‐EGFP (Control, *n* = 6 wells) or pCMV‐LOx‐IRES‐EGFP (LOx, *n* = 9 wells; unpaired two‐tailed *t*‐test). (b) Media conditioned for 6 hr by dissociated cultured astrocytes transduced with AVV‐sGFAP‐EGFP (Control, *n* = 18 wells) or AVV‐sGFAP‐LOx‐IRES‐tdTomato (LOx, *n* = 9 wells; unpaired two‐tailed *t*‐test). (c) Amperometric measurement of extracellular lactate tone in organotypic brainstem slices transduced with AVV‐PRSx8EGFP alone (Control, *n* = 14) or in combination with AVV‐sGFAP‐LOx‐IRES‐tdTomato (LOx, *n* = 12; Mann–Whitney *U* rank test). (d) Representative traces of Laconic FRET ratio in dissociated astrocytic cultures expressing AVV‐CMV‐Laconic alone (Control) or in combination with AVV‐sGFAP‐LOx‐IRES‐tdTomato and exposed to pyruvate (10 mM; highlighted application time). (e) Minimum Laconic FRET ratios in control and LOx‐expressing astrocytes evoked by extrusion of intracellular lactate via trans‐acceleration of MCTs (*n* = 62 cells in Control; *n* = 52 cells in LOx; unpaired two‐tailed *t*‐test). (f) Representative trace of an amperometric recording in organotypic brainstem slices during pyruvate‐induced trans‐acceleration. (g) Trans‐acceleration and (h) depletion amplitude, respectively, following pyruvate (10 mM) exposure in slices transduced with AVV‐PRSx8EGFP alone (Control, *n* = 11) or in combination with AVV‐sGFAP‐LOx‐IRES‐tdTomato (LOx, *n* = 12); unpaired two‐tailed *t*‐test (g) or Mann–Whitney *U* rank test (h) [Color figure can be viewed at wileyonlinelibrary.com]

The effect of LOx expression on release of lactate was assessed in HEK293 cells and in primary astrocytes. Expression of a reporter fluorophore confirmed that the majority of cells were expressing the transgenes (Figure S2a,b). Concentration of lactate in the conditioned extracellular media was significantly decreased by LOx (33.2% in HEK293 cells and 15.4% in astrocytes), reflecting limited constitutive lactate release (Figure 2a,b). In line with this, amperometric recordings revealed that expression of LOx by astrocytes in organotypic slices significantly decreased lactate tone when compared to control slices (Figure 2c).

### 3.3 LOx expression reduces the intracellular lactate pool in vitro

The change in intracellular lactate was measured with confocal Laconic imaging while forcing lactate depletion via pyruvate‐evoked trans‐acceleration (Mächler et al., 2016; San Martín et al., 2013; Zuend et al., 2020). As expected, bath application of pyruvate (10 mM) evoked a substantial drop in the intracellular concentration of lactate in cultured dissociated astrocytes which was significantly decreased in LOx‐expressing cultures (Figure 2d,e).

Trans‐acceleration in cultured brainstem slices evoked an immediate and distinct peak in the extracellular concentration of lactate in all slices (Figure 2f). The net amplitude of trans‐acceleration‐driven lactate release was significantly reduced by 65.8% in slices transduced with AVV‐sGFAP‐LOx‐IRES‐tdTomato (Figure 2g). Pyruvate‐driven lactate release was followed by a transient decrease in lactate concentration (depletion amplitude; Figure 2f) which is thought to reflect the depletion of the readily available intracellular lactate pool that normally overflows as constitutive release. The drop from baseline lactate to the following minimum lactate release was reduced by 78.5% in slice cultures containing LOx‐expressing astrocytes, suggesting, again, a diminished intra‐astrocytic lactate pool (Figure 2h).

### 3.4 In vivo effects of LOx expression in astrocytes of dorsal hippocampus

**FIGURE 3:**
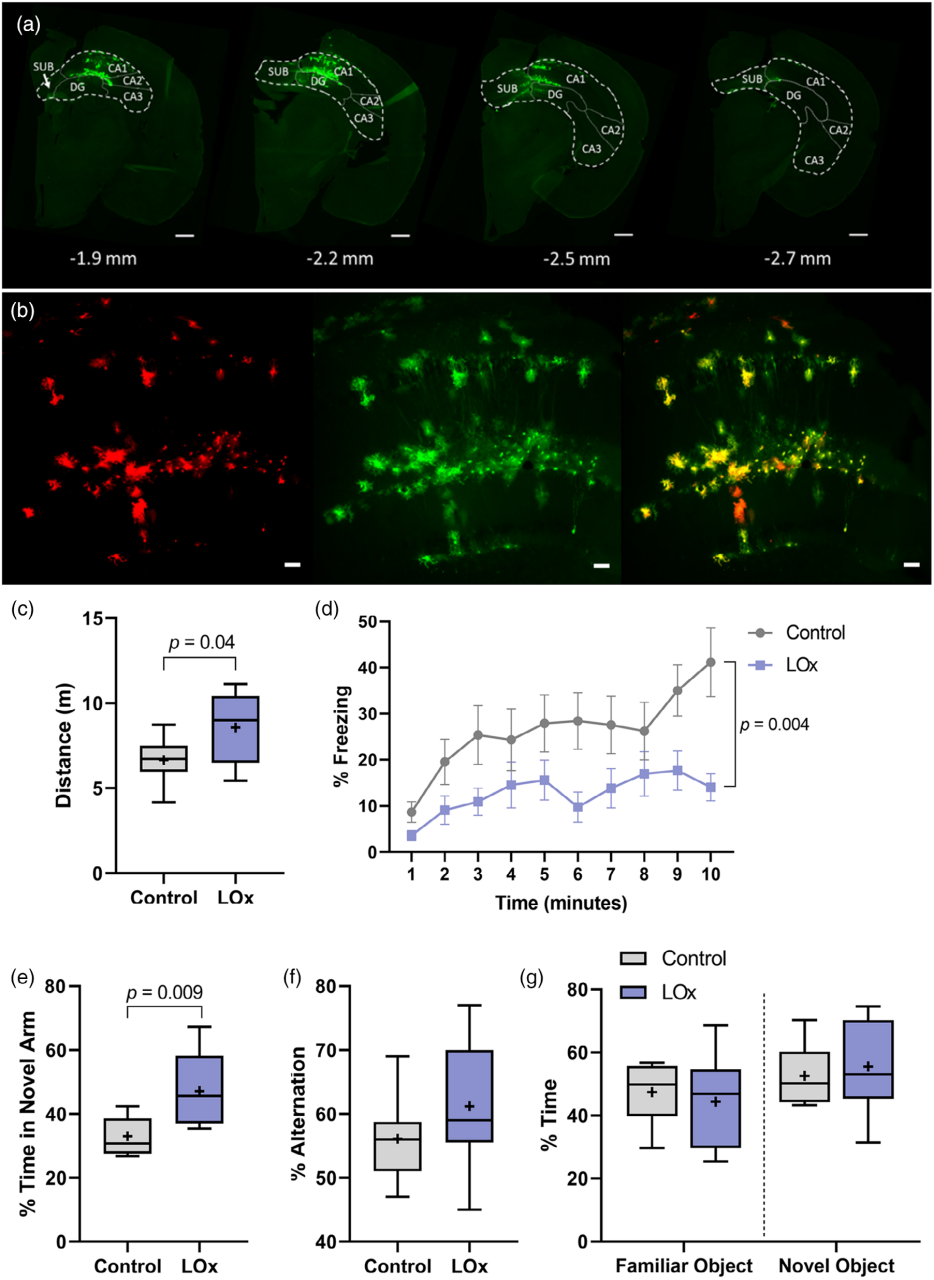
Expression of lactate oxidase (LOx) in hippocampal astrocytes leads to novelty‐induced activity. (a) Serial coronal sections of the mouse brain with control vector (LVV‐sGFAP‐IRES‐tdTomato) injection in the hippocampus and stained with anti‐RFP (green); scale bars 500 μm. (b) Representative images of hippocampal sections with tdTomato^+^ astrocytes (red) co‐stained with anti‐RFP (green); scale bars – 50 μm. (c) In the holeboard test mice expressing LOx in hippocampal astrocytes manifested increased activity compared to control LVV injected mice; (d) During habituation to the trace fear conditioning chamber, mice expressing LOx in hippocampal astrocytes showed less freezing in response to a novel environment; *p < 0.05, *n* = 8–9. (e) Hippocampal LVV‐sGFAP‐LOx‐IRES‐tdTomato injections improved the performance in the task for spatial recognition memory in the Y maze task, with no change in the y‐maze spontaneous alternation task (f) or the novel object recognition test (g); **p < 0.01, *n* = 5–9 [Color figure can be viewed at wileyonlinelibrary.com]

Analysis of expression of tdTomato indicated that LVV‐sGFAP‐LOx‐IRES‐tdTomato transduced astrocytes within a limited area of the dorsal hippocampus (Figure 3a). Using the anti‐RFP antibody, we observed a wider spread of transgene expression (Figure 3b). Expression driven by this LVV was selective for astrocytes as no positive staining was seen in neurones or microglia (Figures S4–S5).

Expression of LOx in hippocampal astrocytes did not alter general activity of mice in the open field test, including central, peripheral or rearing activities (Figure S6). No effects of LOx expression were observed on anxiety‐like behavior in the EPM (Figure S7). Compared to control mice, mice expressing LOx in astrocytes exhibited a significantly increased locomotor activity in the hole board test without altering number of head dips, suggesting elevated novelty‐induced activity (Figure 3c, Figure S7). In addition, mice expressing LOx showed significantly less freezing behavior during the habituation session in the trace fear conditioning test, indicative of elevated reactivity to unfamiliar environment (Figure 3d). When we assessed the effects of LOx on learning and memory, no significant changes were seen in spontaneous alterations in the Y maze test, the novel object recognition test or the context‐ or cue‐dependent conditional freezing in the trace fear conditioning test (Figure 3f,g; Figure S8). In contrast, compared to control mice, mice expressing LOx demonstrated a superior spatial recognition memory in the Y maze (Figure 3e). Collectively, our findings suggest that expression of LOx in hippocampal astrocytes led to increased responsiveness to novelty in mice that could facilitate some types of recognition memory.

## 4 DISCUSSION

**FIGURE 4:**
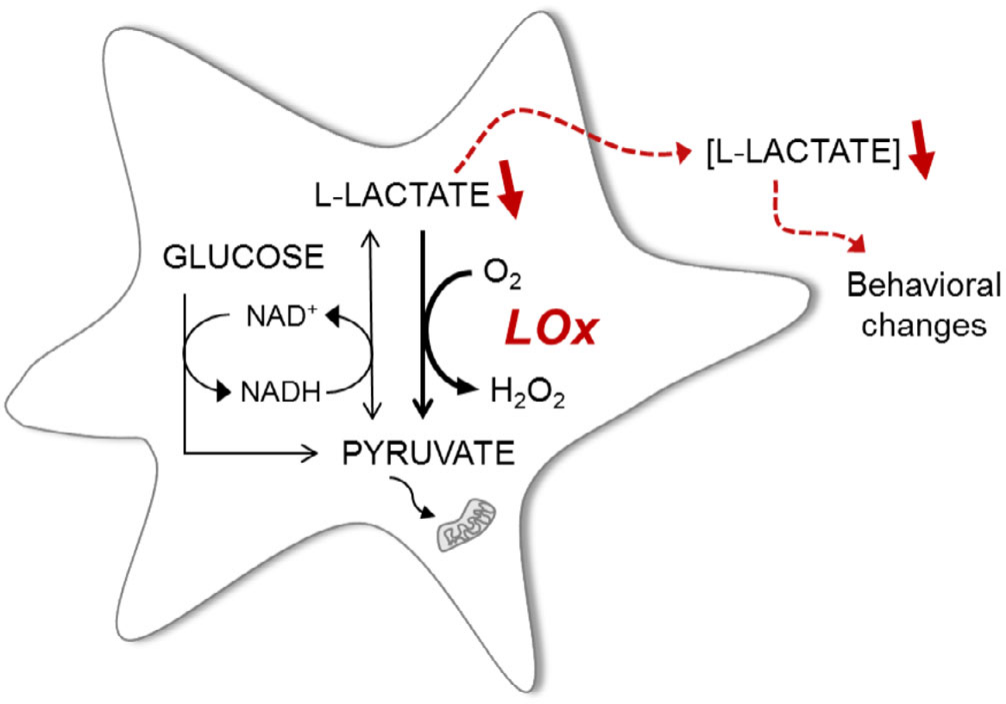
Viral vector‐mediated expression of lactate oxidase (LOx) in astrocytes as a novel tool for accelerated breakdown of intracellular lactate. Constitutive activity of LOx breaks down lactate produced through glycolysis in astrocytes and, consequently, decreases intra‐astrocytic lactate concentration and subsequent release to the extracellular space. This has implications for constitutive as well as stimulus‐evoked lacatate release and for metabolic and signaling roles of lactate in the brain [Color figure can be viewed at wileyonlinelibrary.com]

The release of lactate in the brain is not only dependent on its production rate but astrocytes have the ability to retain higher intracellular lactate levels as a readily releasable pool which is available for stimulus‐evoked transient release (Mächler et al., 2016; Sotelo‐Hitschfeld et al., 2015). Here, we report the validation and application of a novel viral vector‐based molecular approach, designed to limit the intra‐astrocytic lactate pool without directly interfering with astrocytic lactate production or release mechanisms (Figure 4). We have confirmed in vitro that LOx is enzymatically active and changes lactate dynamics in dissociated cultured astrocytes and organotypic brainstem slices. Astrocyte‐specific expression of LOx reduced the constitutive release of lactate, as well as that evoked using a trans‐acceleration protocol. Although trans‐acceleration of MCTs has been recurrently used in vitro and in vivo to deplete cells of lactate, this effect comes at the expense of cellular overload with pyruvate, which could result in lactate dehydrogenase‐mediated conversion of pyruvate to lactate, increasing cytosolic lactate concentration. In order to restrict lactate formation following pyruvate application, the glucose concentration in the superfusion buffer was reduced so that the generation of NADH through glycolysis was limited and, with it, the activity of lactate dehydrogenase (San Martín et al., 2013). We found that trans‐accelerated lactate extrusion was substantially decreased in organotypic brainstem slices that contained both neurones and astrocytes. However, as LOx expression was targeted to astrocytes, our findings suggest that limiting the lactate pool selectively in astrocytes makes a significant impact on lactate dynamics in the integrated brain environment.

The degradation of lactate to pyruvate by LOx results in production of hydrogen peroxide (Maeda‐Yorita, Aki, Sagai, Misaki, & Massey, 1995; Stoisser, Brunsteiner, Wilson, & Nidetzky, 2016) (Figure 4). While peroxide is a potentially harmful molecule, astrocytes have been shown to exhibit a high glutathione content and a robust capacity for glutathione‐dependent detoxification (Dringen, Brandmann, Hohnholt, & Blumrich, 2015; Liu, Teschemacher, & Kasparov, 2017). In order to minimize the potentially cytotoxic effects of ROS‐mediated cellular damage or overexpression of a foreign protein product on astrocytic metabolism, we optimized vector titers to express LOx at the levels that did not seem to lead to any untoward consequences. Our in vivo observations do not suggest overt toxic effects on astrocytes either but future studies will perform a more detailed evaluation of possible ROS‐mediated cellular effects of LOx expression.

The behavioral effects of astrocyte‐restricted expression of LOx in the dorsal hippocampus suggest that decreased release of lactate by hippocampal astrocytes increased responsiveness to novelty and did not affect anxiety in mice. These changes are consistent with the different functions of the dorsal vs. ventral hippocampus. It has been shown that the processing of novelty and spatial information is predominantly associated with the dorsal sub‐regions of the hippocampus, while emotionality and anxiety have been preferentially attributed to the ventral areas of the hippocampus (Bannerman et al., 2014). This also concords with studies showing that peripheral lactate administration did not alter baseline anxiety in rodents (Karnib et al., 2019).

Our behavioral results are consistent with numerous findings from humans, nonhuman primates, and rodents that the hippocampus plays a central role in novelty detection, and the activity of the hippocampal circuits increases in response to novel stimuli (Murty, Ballard, Macduffie, Krebs, & Adcock, 2013). On the one hand, increased activity in the hole board test is in line with increased exploratory activity in LOx expressing animals. On the other hand, as LOx expression led to low freezing during the habituation stage in TFC, one could propose that decreased lactate release by hippocampal astrocytes might affect habituation to the novel environment in mice. Notably, a recent study reports that selective chemogenetic activation of striatal astrocytes produced “acute behavioral hyperactivity and disrupted attention” and that thrombospodin‐1 in astrocytes was required for manifestation of the behavioral phenomena (Nagai et al., 2019). It is tempting to speculate that our findings point to another possible pathway that could be at play in the neuron‐astrocyte interactions underlying the higher functions of the hippocampus. Furthermore, one could hypothesize that given the short period of time during which the mice were behaviorally evaluated, our data suggest a signaling rather than metabolic role for lactate in the tests used. Future studies using viral vector‐mediated LOx expression will help shed more light on the mechanisms and processes whereby astrocyte lactate is involved in inter‐cellular communications in the brain.

In conclusion, astrocyte‐specific expression of LOx to limit intracellular lactate pools represents a novel approach to elucidate the functions of lactate in different brain cells. This approach allows for manipulating lactate dynamics in chronic studies to elucidate the roles of lactate in intercellular signaling and/or energy metabolism. We anticipate that the present study will facilitate a more accurate and nuanced understanding of how astrocytic lactate contributes to the central networks in various physiological and pathological contexts (Figure 4).

## CONFLICT OF INTEREST

The authors declare no potential conflict of interest.

## Supporting information


**Figure S1** Cassette for expression of LOx in cell lines and in astrocytes in vitro and in vivo. (a) LOx and control constructs in plasmids for CMV‐driven expression in acutely transfected HEK293 cells – *CMV‐LOx‐IRES‐EGFP* and *CMV‐IRES‐EGFP*, respectively. Enhanced green fluorescent protein (EGFP) serves as expression marker and is preceded by an internal ribosomal entry site (IRES). (b) Construct in adenoviral vector (AVV) backbone for expression of LOx and fluorescent marker in astrocytes in vitro*—AVV‐sGFAP‐LOx‐IRES‐tdTomato*. A transcriptionally enhanced short glial fibrillary acidic protein promoter (sGFAP; Liu, Paton, & Kasparov, 2008) restricts expression to astrocytes. tdTomato was used as fluorescent marker. (c) Cassettes in lentiviral vector (LVV) backbone for expression of LOx and control fluorescent marker in astrocytes in vivo (Duale, Kasparov, Paton, & Teschemacher, 2005)—*LVV‐sGFAP‐LOx‐IRES‐tdTomato* and *LVV‐sGFAP‐IRES‐tdTomato*, respectively. A Woodchuck Hepatitis Virus posttranscriptional regulatory element (WPRE) was included to stabilize expression levels.
**Figure S2.** Representative images of co‐expression of fluorescent reporters with LOx in vitro. (a) EGFP expression in CMV‐LOx‐IRES‐EGFP transfected HEK293 cells. (b) Dissociated cultures of rat astrocytes transduced with AVV‐sGFAP‐LOx‐IRES‐tdTomato. (c) Organotypic brainstem slice cultures transduced with AVV‐sGFAP‐LOx‐IRES‐tdTomato. tdTomato signal amplified by anti‐RFP staining in (b) and (c). Scale bars 50 μm.
**Figure S3.** LOx expression does not increase oxidative stress in astrocytes. Fluorescence intensity of H_2_DCFDA compared to baseline in dissociated cultured astrocytes transduced with AVV‐sGFAP‐LOx‐IRES‐tdTomato at MOI 15 (LOx, *n* = 9 wells). Non‐transduced astrocytes were used as negative control (Control, *n* = 9 wells), astrocytes exposed to H_2_O_2_ for 10 min were used as positive control (H_2_O_2_, *n* = 5 wells). ANOVA followed by Bonferroni's Multiple Comparison post hoc test.
**Figure S4.** Astrocyte‐specific expression in hippocampus in vivo following transduction with LVV‐sGFAP‐LOx‐IRES‐tdTomato. (a) Representative images of tdTomato^+^ (red) astrocytes co‐stained with anti‐S100β (green) antibody. (b) Representative images of tdTomato^+^ (red) astrocytes co‐stained with anti‐GFAP (green) antibody. Scale bars 10 μm.
**Figure S5.** Lack of neuronal and microglial expression in vivo following transduction with LVV‐sGFAP‐LOx‐IRES‐tdTomato. Representative images of tdTomato^+^ (red) astrocytes co‐stained with anti‐IBA1 (green) or anti‐NeuN (magenta) antibody. Scale bar 10 μm.
**Figure S6.** No effects of LOx expression in hippocampal astrocytes on locomotor activity. Locomotor activity was measured during 30 min in the open field. Total activity (a), total rearing (b), central (c) and peripheral (d) locomotor activities were not different between animals expressing LOx and controls; *p* > 0.05, *n* = 8–9.
**Figure S7.** No effects of LOx expression in hippocampal astrocytes on anxiety‐related or exploratory behaviors. Anxiety‐like behaviors were evaluated in the elevated plus maze (EPM). There was no difference in percent of time spent in the open arms (a) and the distance traveled (b) between control and LOx groups. (c) Exploratory behavior was examined in in the holeboard test. There was no significant difference in the number of head dips between control and LOx groups. *p* > 0.05, *n* = 8–9.
**Figure S8.** No effects of LOx expression in hippocampal astrocytes on trace fear conditioning. (a) No difference in freezing behavior was found between control and LOx groups during the training session, where a 20 sec tone, followed by a 2 sec shock, was delivered four times as indicated. No difference was observed 24 hr after the training session in context‐dependent memory (b), nor in cue‐dependent memory (c); p 0.05, *n* = 8–9.
**Table S1**. Antibody information

## Data Availability

Data available on request from the authors.
