# Supplementary material for: Reducing l‐lactate release from hippocampal astrocytes by intracellular oxidation increases novelty induced activity in mice
Source: Glia. 2021 Jan 5;69(5):1241–50. doi: 10.1002/glia.23960 (PMC8576740; doi:10.1002/glia.23960)
Supplement: Supplementary file 1 — Figure S1 Cassette for expression of LOx in cell lines and in astrocytes in vitro and in vivo. (a) LOx and control constructs in plasmids for CMV‐driven expression in acutely transfected HEK293 cells – CMV‐LOx‐IRES‐EGFP and CMV‐IRES‐EGFP, respectively. Enhanced green fluorescent protein (EGFP) serves as expression marker and is preceded by an internal ribosomal entry site (IRES). (b) Construct in adenoviral vector (AVV) backbone for expression of LOx and fluorescent marker in astrocytes in vitro—AVV‐sGFAP‐LOx‐IRES‐tdTomato. A transcriptionally enhanced short glial fibrillary acidic protein promoter (sGFAP; Liu, Paton, & Kasparov, 2008) restricts expression to astrocytes. tdTomato was used as fluorescent marker. (c) Cassettes in lentiviral vector (LVV) backbone for expression of LOx and control fluorescent marker in astrocytes in vivo (Duale, Kasparov, Paton, & Teschemacher, 2005)—LVV‐sGFAP‐LOx‐IRES‐tdTomato and LVV‐sGFAP‐IRES‐tdTomato, respectively. A Woodchuck Hepatitis Virus posttranscriptional regulatory element (WPRE) was included to stabilize expression levels. Figure S2. Representative images of co‐expression of fluorescent reporters with LOx in vitro. (a) EGFP expression in CMV‐LOx‐IRES‐EGFP transfected HEK293 cells. (b) Dissociated cultures of rat astrocytes transduced with AVV‐sGFAP‐LOx‐IRES‐tdTomato. (c) Organotypic brainstem slice cultures transduced with AVV‐sGFAP‐LOx‐IRES‐tdTomato. tdTomato signal amplified by anti‐RFP staining in (b) and (c). Scale bars 50 μm. Figure S3. LOx expression does not increase oxidative stress in astrocytes. Fluorescence intensity of H2DCFDA compared to baseline in dissociated cultured astrocytes transduced with AVV‐sGFAP‐LOx‐IRES‐tdTomato at MOI 15 (LOx, n = 9 wells). Non‐transduced astrocytes were used as negative control (Control, n = 9 wells), astrocytes exposed to H2O2 for 10 min were used as positive control (H2O2, n = 5 wells). ANOVA followed by Bonferroni's Multiple Comparison post hoc test. Figure S4. Astrocyte‐speci [file GLIA-69-1241-s001.docx]

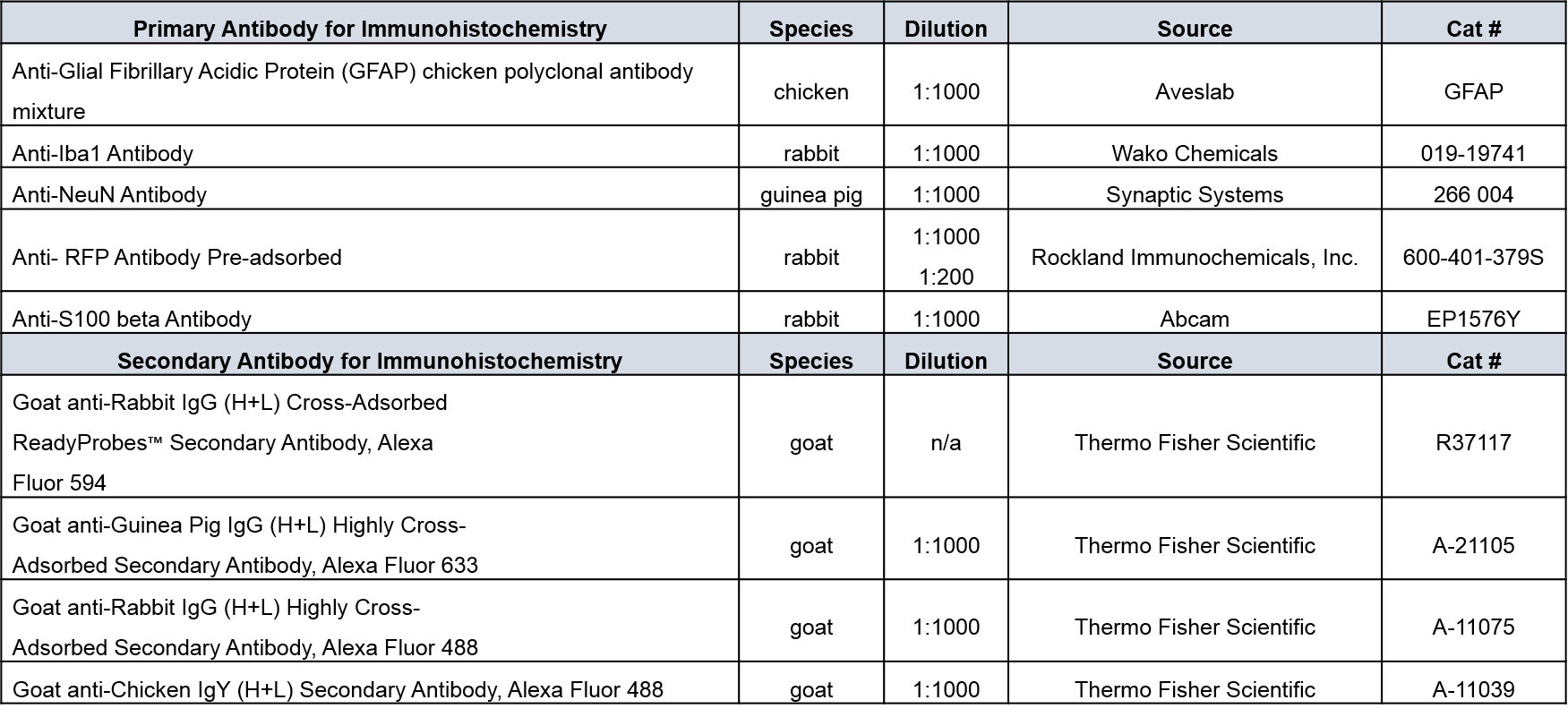


**Suppl Table 1.** Antibody information


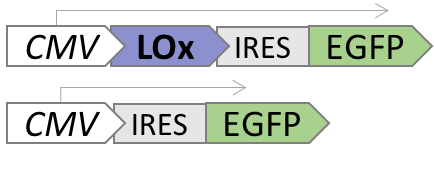

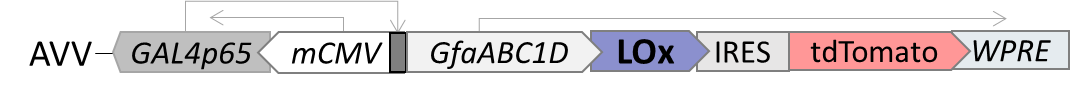

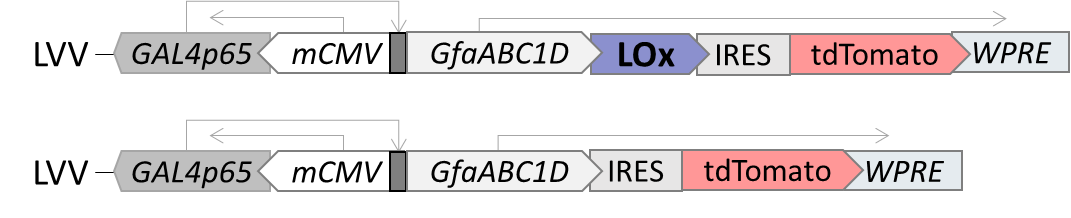


**(a)**

**(c)**

**(b)**

**Suppl Figure 1.**

**(c)**


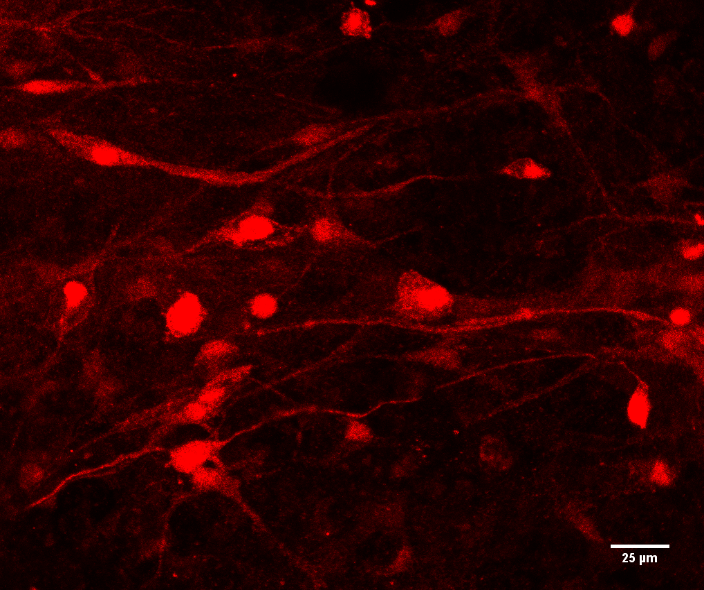

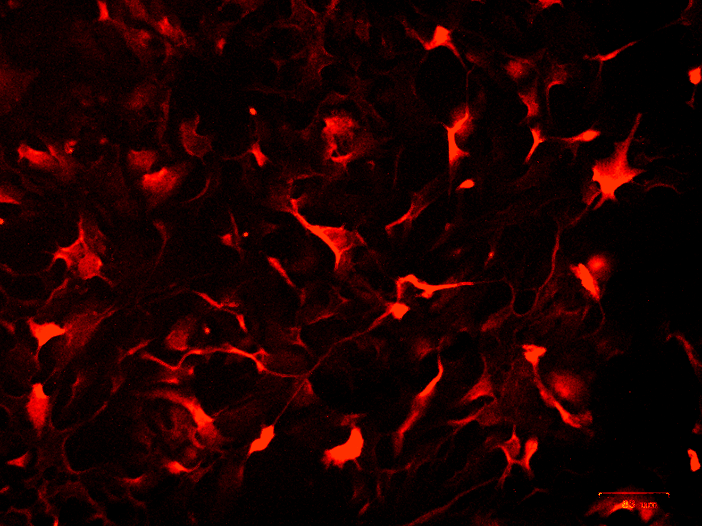


**(b)**

**(a)**


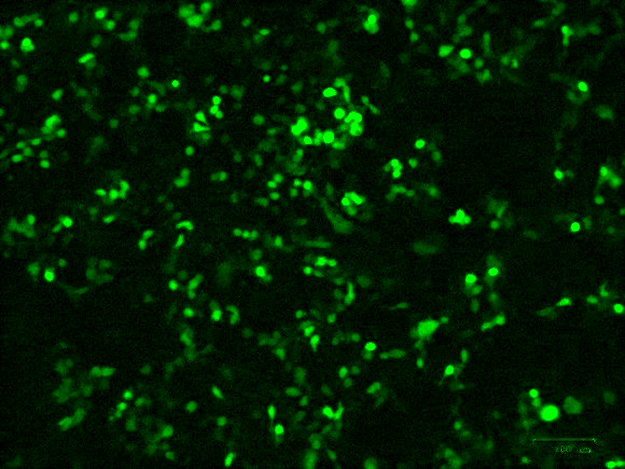


**Suppl Figure 2.**


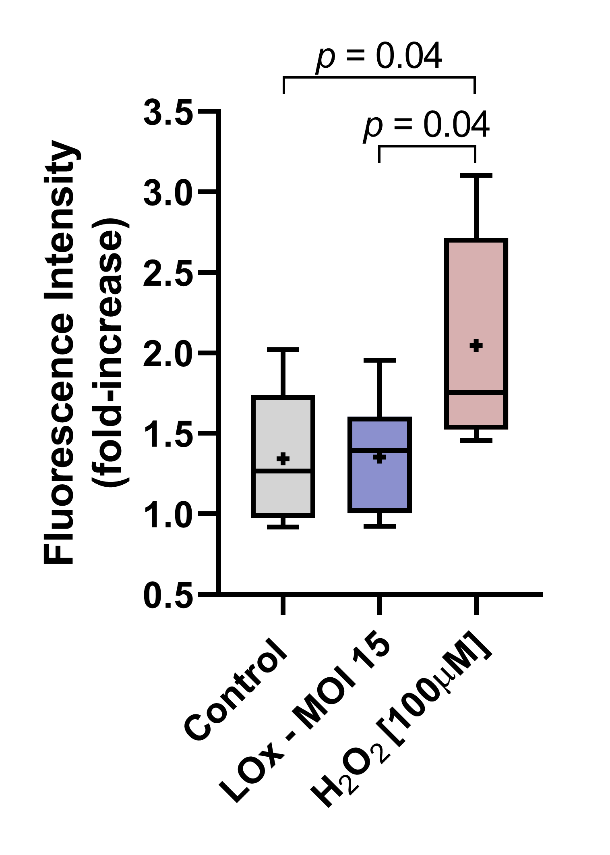


**Suppl Figure 3.**

**Suppl Figure 4.**


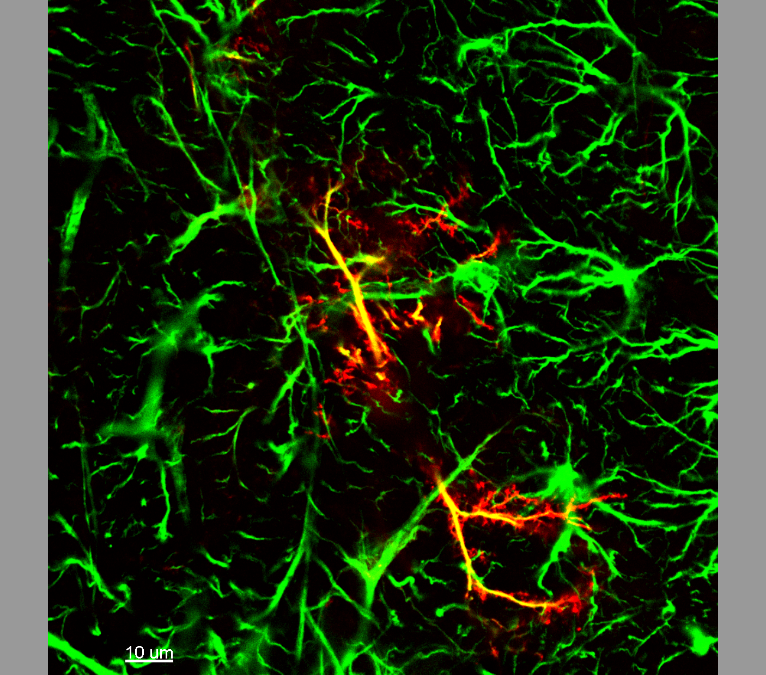

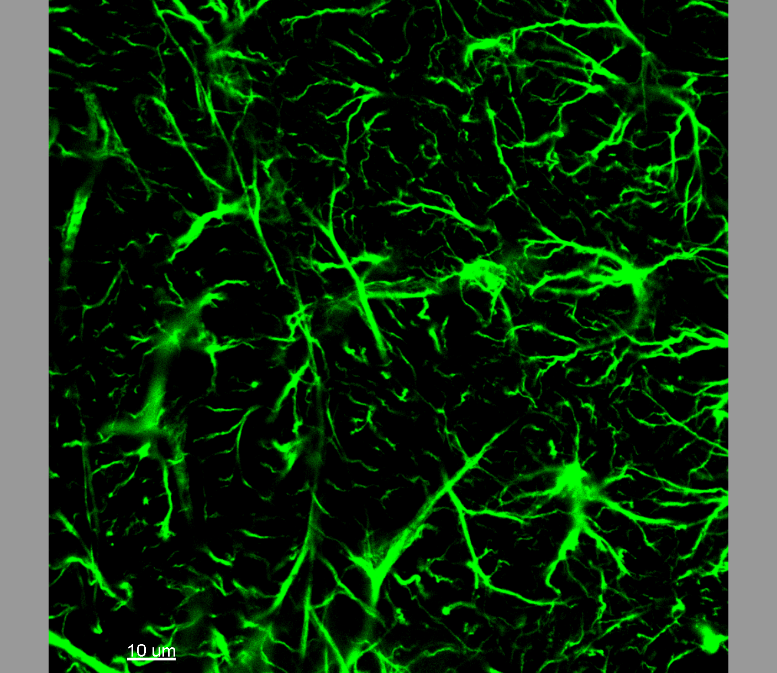

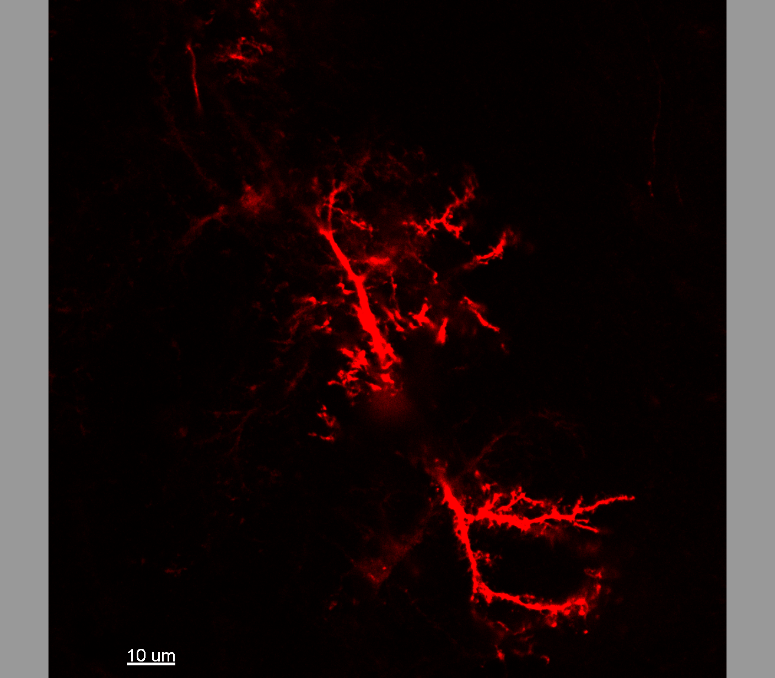

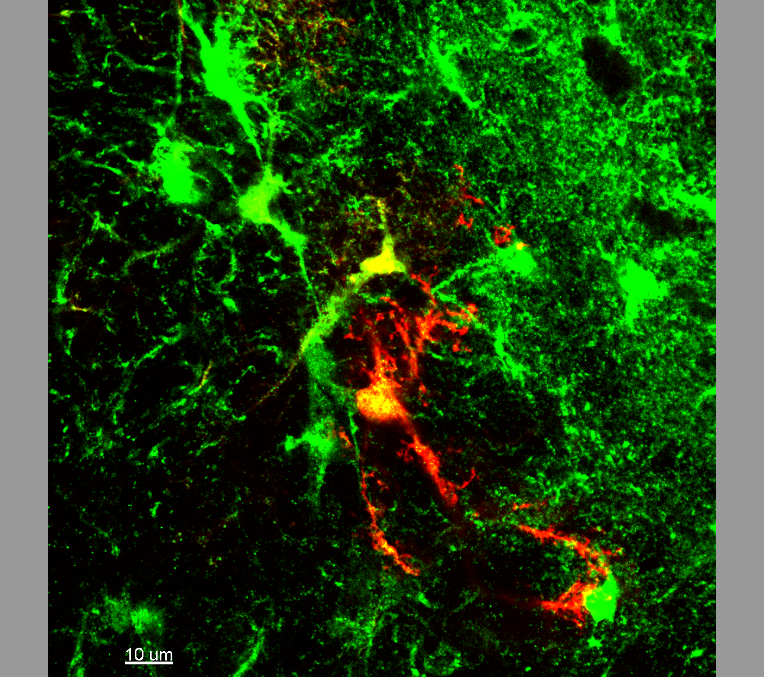

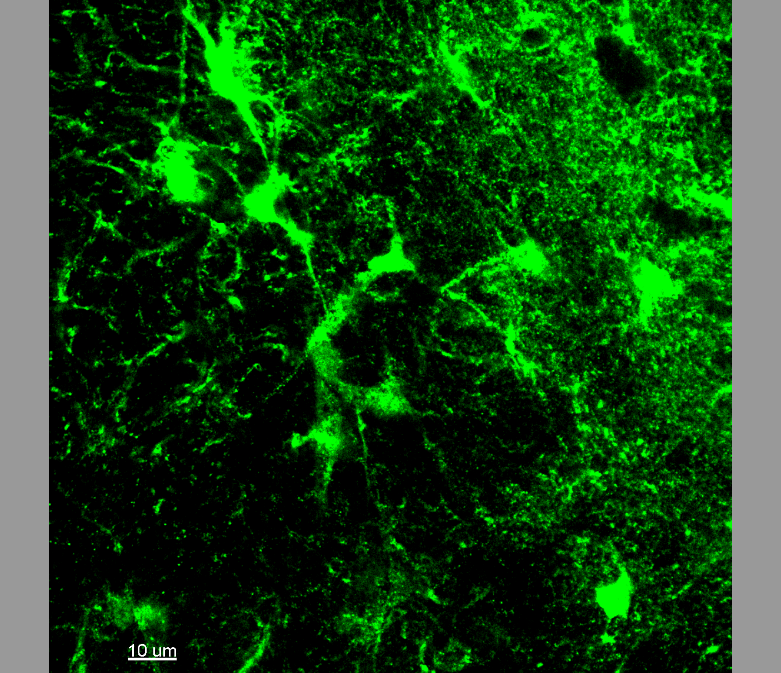

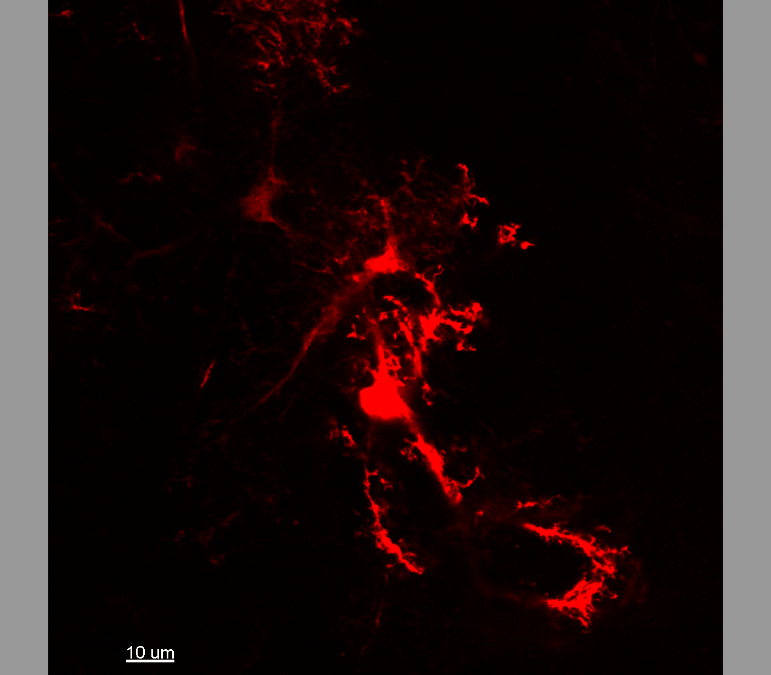


**(b)**

**(a)**

**Merge**

**tdTomato**

**tdTomato**

**Merge**

**GFAP**

**Merge**

**NeuN**

**tdTomato**

**IBA1**


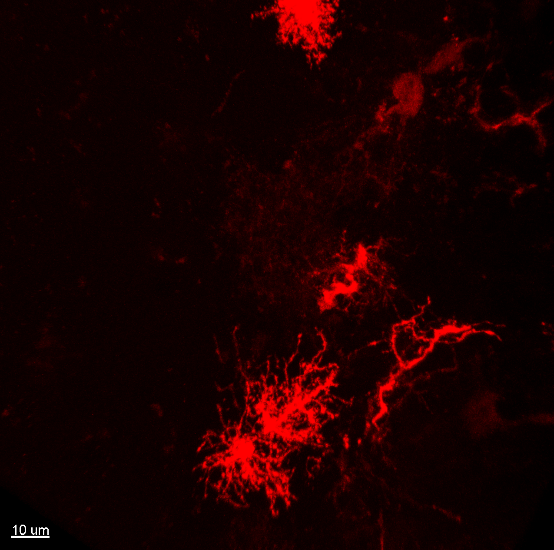

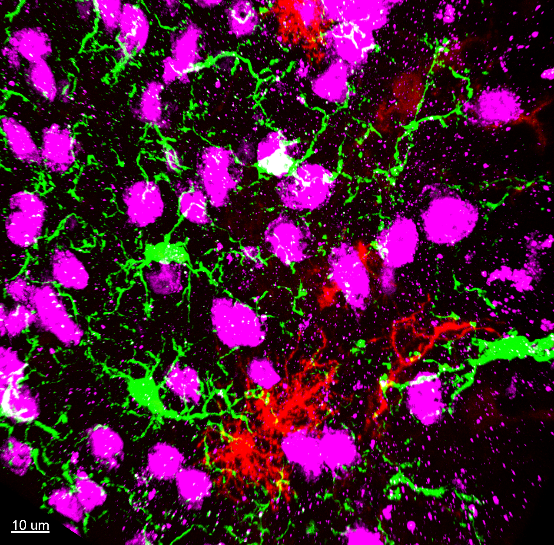

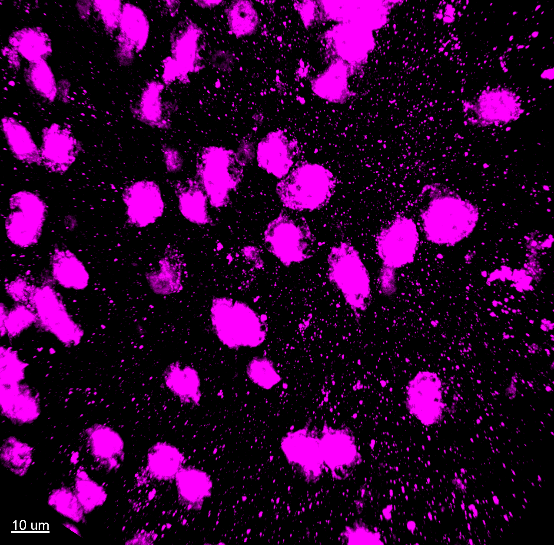

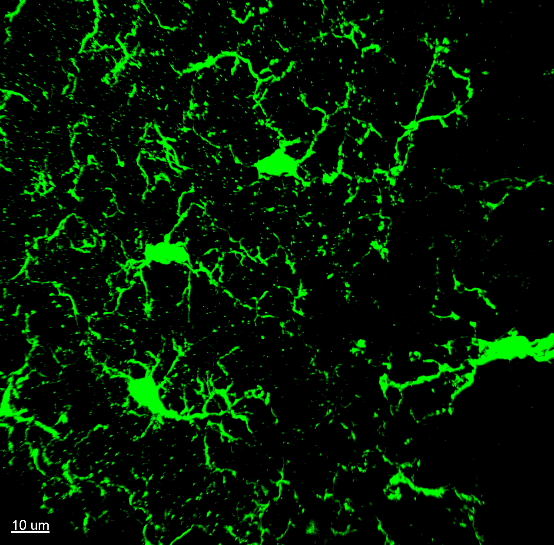


**Suppl Figure 5.**


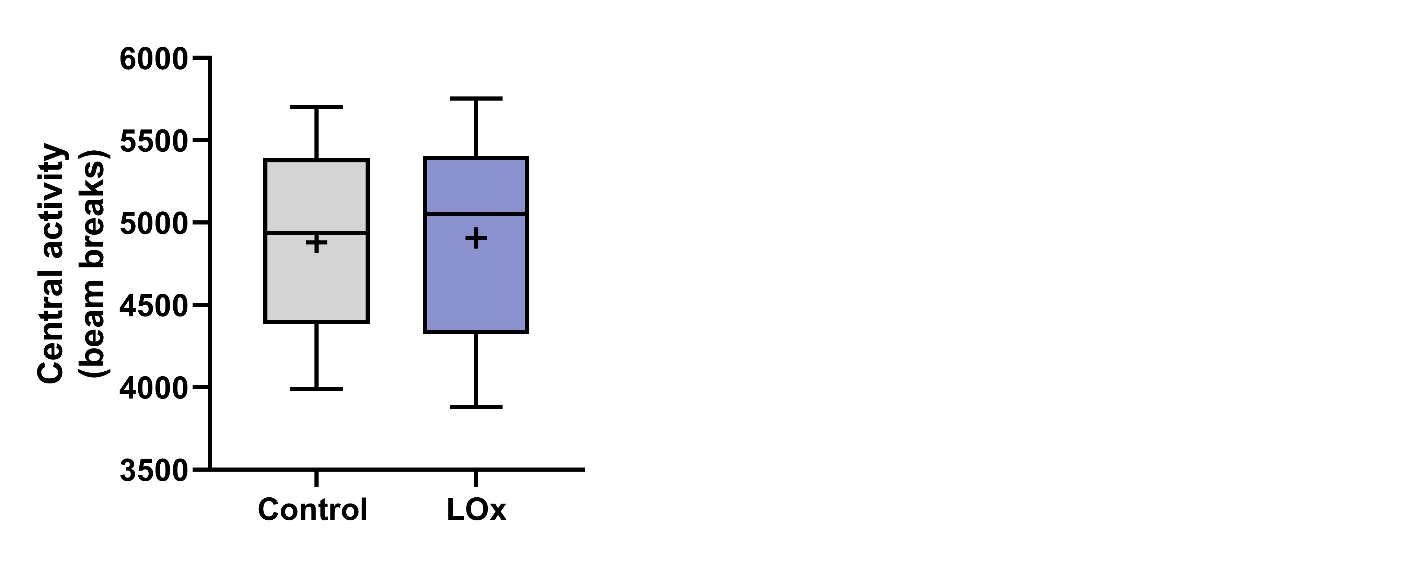

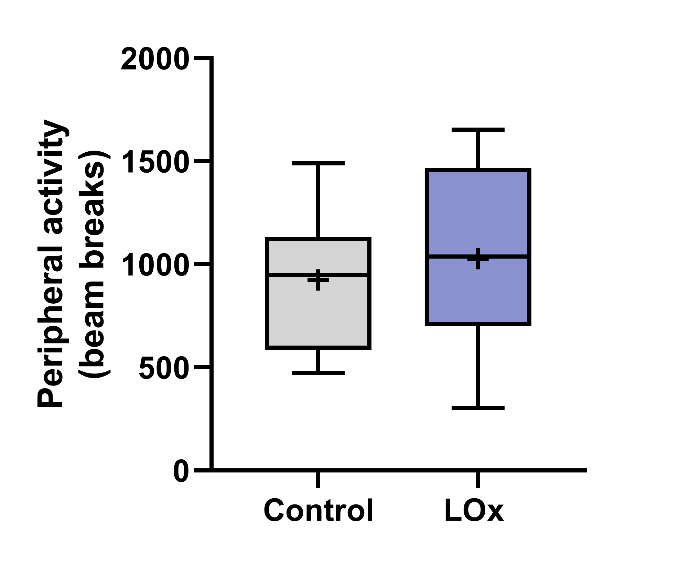

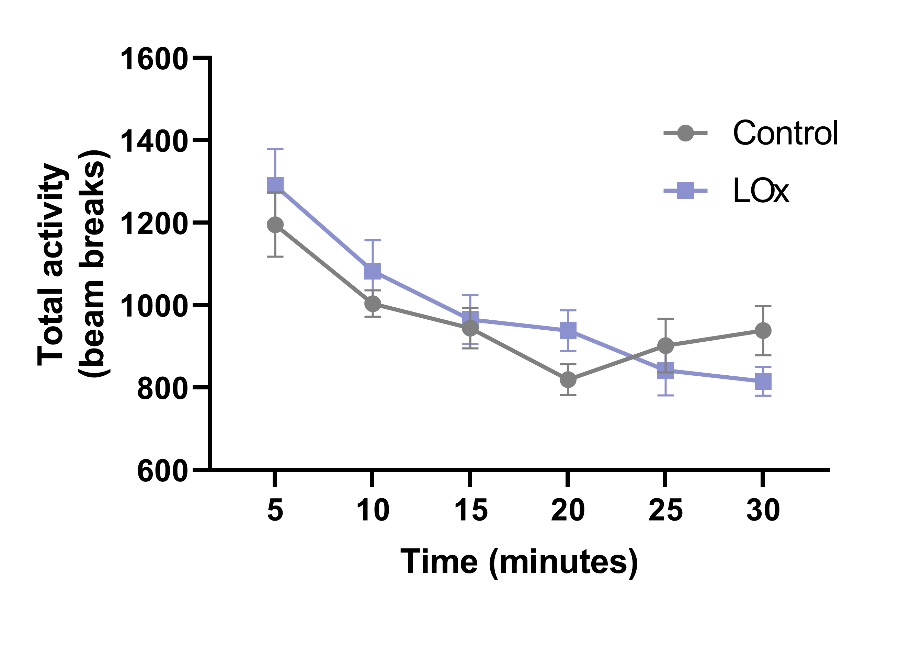

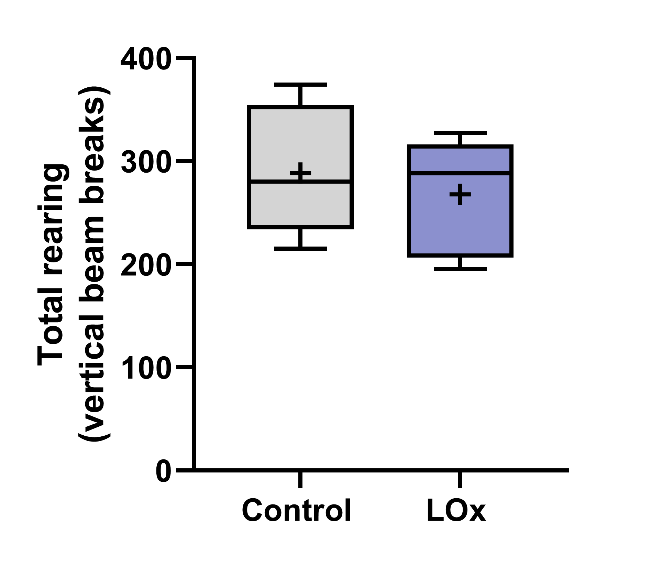


**(c)**

**(d)**

**(a)**

**(b)**

**Suppl Figure 6.**


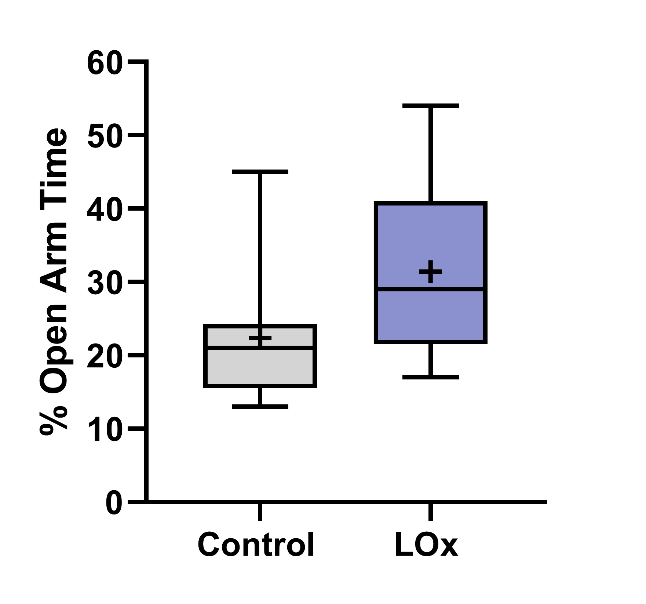

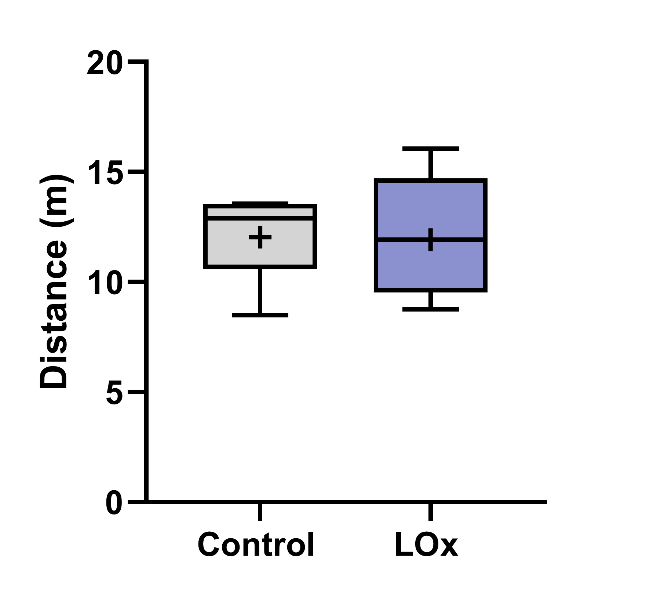

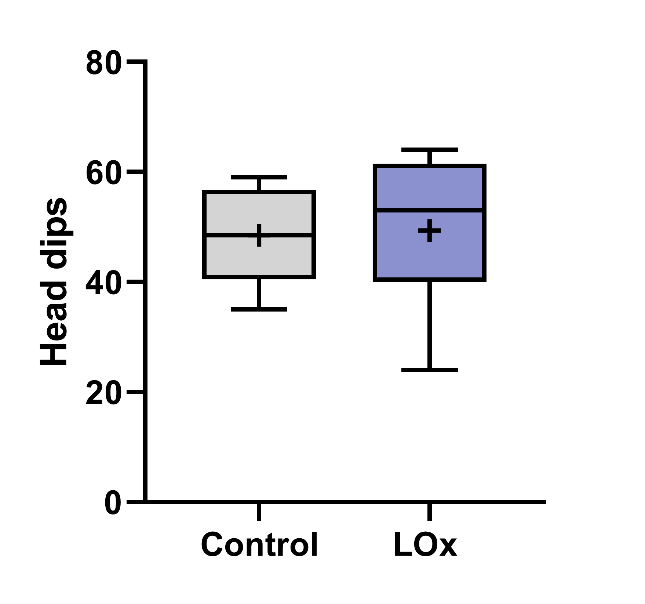


**(a)**

**(b)**

**(c)**

**Suppl Figure 7.**


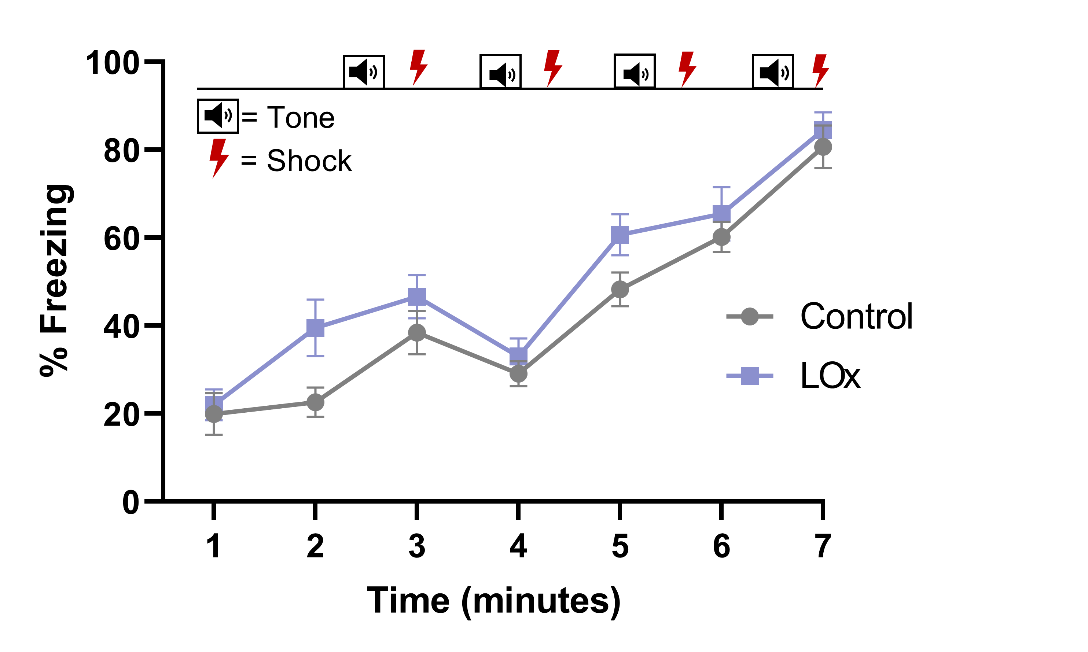

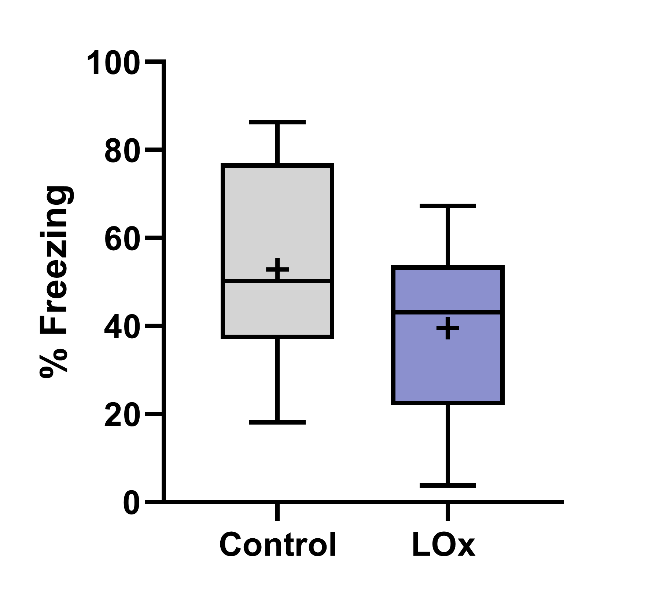

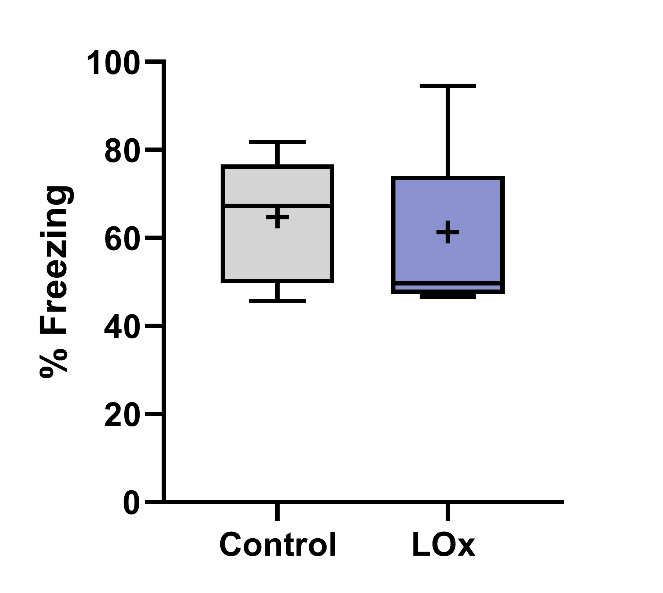


**(a)**

**(c)**

**(b)**

**Suppl Figure 8.**
